# The Synthetic Peptide LyeTx I mn∆K, Derived from *Lycosa erythrognatha* Spider Toxin, Is Active against Methicillin-Resistant *Staphylococcus aureus* (MRSA) In Vitro and In Vivo

**DOI:** 10.3390/antibiotics13030248

**Published:** 2024-03-08

**Authors:** Ana Paula Gonçalves Coelho Vieira, Amanda Neves de Souza, William Gustavo Lima, Julio Cesar Moreira Brito, Daniela Carolina Simião, Lucas Vinícius Ribeiro Gonçalves, Lídia Pereira Barbosa Cordeiro, Denise de Oliveira Scoaris, Simone Odília Antunes Fernandes, Jarbas Magalhães Resende, Burkhard Bechinger, Rodrigo Moreira Verly, Maria Elena de Lima

**Affiliations:** 1 Faculdade de Saúde Santa Casa de Belo Horizonte, Programa de Pós-Graduação *Stricto Sensu* em Medicina e Biomedicina, Belo Horizonte 30150-240, Brazil; anapaula.goncalvescoelho@gmail.com (A.P.G.C.V.); 027796@faculdadesantacasabh.edu.br (W.G.L.); 026992@faculdadesantacasabh.edu.br (L.V.R.G.); 2 Departamento de Química, FACET, Universidade Federal dos Vales do Jequitinhonha e Mucuri (UFVJM)—Campus JK, Diamantina 39100-000, Brazil; amanda.neves@ufvjm.edu.br; 3 Institut de Chimie, Centre National de la Recherche Scientifique, UMR7177, Université de Strasbourg, 67070 Strasbourg, France; bechinge@unistra.fr; 4 Fundação Ezequiel Dias (FUNED), Belo Horizonte 30510-010, Brazil; julio.brito@funed.mg.gov.br (J.C.M.B.); denise.scoaris@funed.mg.gov.br (D.d.O.S.); 5 Laboratório de Radioisótopos, Departamento de Análises Clínicas e Toxicológicas, Faculdade de Farmácia—Campus Pampulha, Universidade Federal de Minas Gerais (UFMG), Belo Horizonte 31270-901, Brazil; danielacs@ufmg.br (D.C.S.); simoneodilia@yahoo.com.br (S.O.A.F.); 6 Departamento de Química, Instituto de Ciências Exatas, Universidade Federal de Minas Gerais (UFMG), Belo Horizonte 31270-901, Brazil; lidiabarbosa0601@ufmg.br (L.P.B.C.); jmr@ufmg.br (J.M.R.); 7 Institut Universitaire de France (IUF), 75005 Paris, France

**Keywords:** antimicrobial peptides, multidrug-resistant pathogens, drug development, skin infections, action on membrane

## Abstract

The urgent global health challenge posed by methicillin-resistant *Staphylococcus aureus* (MRSA) infections demands effective solutions. Antimicrobial peptides (AMPs) represent promising tools of research of new antibacterial agents and LyeTx I mn∆K, a short synthetic peptide based on the *Lycosa erythrognatha* spider venom, is a good representative. This study focused on analyzing the antimicrobial activities of LyeTx I mn∆K, including minimum inhibitory and bactericidal concentrations, synergy and resensitization assays, lysis activity, the effect on biofilm, and the bacterial death curve in MRSA. Additionally, its characterization was conducted through isothermal titration calorimetry, dynamic light scattering, calcein release, and finally, efficacy in a mice wound model. The peptide demonstrates remarkable efficacy against planktonic cells (MIC 8–16 µM) and biofilms (>30% of inhibition) of MRSA, and outperforms vancomycin in terms of rapid bactericidal action and anti-biofilm effects. The mechanism involves significant membrane damage. Interactions with bacterial model membranes, including those with lysylphosphatidylglycerol (LysylPOPG) modifications, highlight the versatility and selectivity of this compound. Also, the peptide has the ability to sensitize resistant bacteria to conventional antibiotics, showing potential for combinatory therapy. Furthermore, using an in vivo model, this study showed that a formulated gel containing the peptide proved superior to vancomycin in treating MRSA-induced wounds in mice. Together, the results highlight LyeTx I mnΔK as a promising prototype for the development of effective therapeutic strategies against superficial MRSA infections.

## 1. Introduction

*Staphylococcus aureus* is a gram-positive coccus, that produces catalase, coagulase, and pyrrolidonyl arylamidase (PYRase). This bacterium is recognized as one of the major infectious disease-causing pathogens in humans. Antimicrobials such as β-lactams are generally used for chemotherapy of these diseases; however, the emergence of methicillin-resistant *S. aureus* (MRSA), which is virtually resistant to all antimicrobials from this class, has posed serious problems [1]. Currently, it is estimated that 11,000 people die each year from MRSA-related infections in the USA, representing nearly half of all fatalities caused by multidrug-resistant (MDR) pathogens in the country [2]. In cases of MRSA infections, few pharmacological options are clinically available, such as vancomycin, mupirocin and daptomycin, which are drugs that have a slow bactericidal effect, significant adverse reactions and are expensive [3]. Thus, the development of new effective and safe therapeutic agents against MRSA-induced infections is imperative to control the public health emergency related to this superbug [4]. 

In this scenario, antimicrobial peptides (AMPs) are highlighted as a promising source of new antimicrobials against MDR pathogens, especially against MRSA [5,6]. The AMPs are components of innate immunity found in various species, from bacteria and fungi to animals and plants [7,8]. There are eight AMPs approved for clinical use by the Food and Drug Administration (FDA) (i.e., gramicidin, colistin, polymyxin B, daptomycin, vancomycin, oritavancin, dalbavancin, and telavancin), all of them are considered “last line” antimicrobials and are active even against MDR specimens [9]. These agents are known to act on the cell membrane of bacterial pathogens stimulating the lysis and release of cytoplasmic contents. Furthermore, they can cross the membrane inhibiting different metabolic pathways in a bacterial cell and can modulate the antibacterial immune response [10,11,12]. AMPs have a series of advantages over conventional therapies such as a rapid bactericidal effect, low potential for inducing resistance, absence of the formation of active residues that can contaminate the environment, and action on MDR isolates [13,14].

One of the main sources of AMPs in nature are animal toxins. In fact, numerous compounds with antimicrobial activity have been isolated from the venom of spiders, scorpions, snakes, frogs, bees, wasps, ants and other animals, highlighting this biological matrix as a potential source for the search for new antimicrobials against MRSA [15,16]. Herein, our group has isolated a peptide containing 25 amino acid residues (H-IWLTALKFLGKNLGKHLAKQQLAKL-NH_2_) from the venom of the Brazilian spider *Lycosa erythrognatha*, popularly known as the “*aranha-lobo*”, “*aranha-de-jardim*”, or “*tarantula*”, which has demonstrated good antimicrobial activity. In vitro studies have shown that the LyeTx I peptide proved to be active against *Escherichia coli* (MIC: 1.41–7.81 μM), *Pseudomonas aeruginosa* (MIC: 2.82 μM), *Acinetobacter baumannii* (MIC: 0.70 μM), *Staphylococcus aureus* (MIC: 0.70–3.79 μM), *Staphylococcus epidermidis* (MIC: 0.70 μM), *Pichia kudriavzevii* (formerly *Candida krusei*) (MIC: 14.23–26.30 μM), *Cryptococcus gattii* (MIC: 2.23 μM), and *Cryptococcus neoformans* (MIC: 7.11–13.20 μM) [17]. However, peptides that have many amino acid residues end up having a restriction on their availability since the synthesis process of this molecules is laborious and expensive, with the costs being directly proportional to the size of the primary sequence. Thus, in order to obtain a smaller molecule while retaining antimicrobial activity, LyeTx I was minimized from the *C*-terminal region by sequentially removing amino acid residues up to the alanine at position 15 [18]. Additionally, this shortened derivative was changed by insertion of a lysine residue at position 5, resulting in a new peptide named LyeTx I mnΔK that contains only 16 amino acid residues (H-IWLTKALKFLGKNLGK-NH_2_). This compound, in addition to presenting a spectrum of activity very similar to that of LyeTx I [18], was able to act on both, planktonic cells and biofilms of *Candida* spp. [19] and carbapenem-resistant *Acinetobacter baumannii* (CRAB) [19]. Moreover, intranasal instillation of LyeTx I mnΔK (1–10 mg/Kg) was able to reduce the pulmonary bacterial load of animals with CRAB-induced lower airway infection, indicating its potential therapeutic use against superbug infections [20]. 

Considering the clinical relevance of MRSA infections and the limited therapeutic arsenal available against this pathogen, and taking into account the good antibacterial activity obtained in vitro and in vivo with LyeTx I mnΔK against MDR bacteria, the use of this compound to treat MRSA infections is promising. In this study, we investigated the therapeutic potential of the LyeTx I mnΔK peptide for non-surgical wounds caused by MRSA in mice, and in addition, we employed biophysical studies to evaluate the membrane binding properties of this peptide, in the presence of *S. aureus* mimetic membranes. 

## 2. Results

### 2.1. LyeTx I mn∆K Shows Good In Vitro Activity against MRSA

The antibacterial activity of LyeTx I mnΔK was evaluated by a broth microdilution assay against MRSA isolated from infected wounds of patients hospitalized in a tertiary hospital in southeast Brazil. As shown in Table 1, LyeTx I mn∆K inhibited the growth of all isolates tested, with MICs ranging from 8 to 32 μM, where the MIC required to inhibit 50% of MRSA isolates (MIC_50_) was 8 µM. The antimicrobial effect observed was mostly bactericidal, and it was shown that LyeTx I mn∆K could kill MRSA cells at concentrations ranging from 16 to 32 μM. Vancomycin, in turn, shows a MIC_50_ of 1 μM and MBCs between 2 and 8 μM (Table 1).

### 2.2. LyeTx I mn∆K Exhibits Rapid Bactericidal Effects on Cells in Logarithmic Growth of MRSA

**Figure 1:**
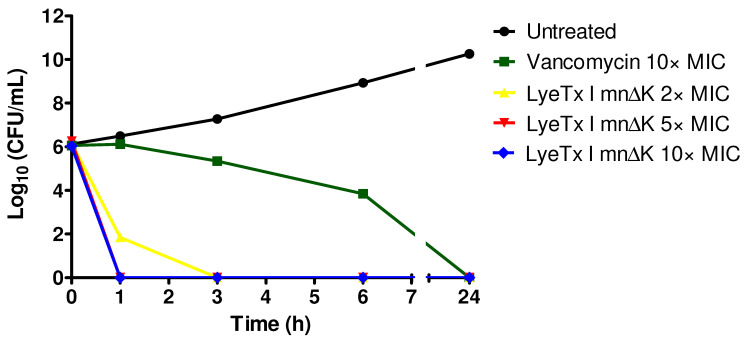
Time-kill curves of LyeTx I mn∆K and vancomycin against a suspension of methicillin-resistant *Staphylococcus aureus* (MRSA) on logarithmic growth phase (10^6^ CFU/mL). MIC: minimum inhibitory concentration.

The bactericidal action in function of time of LyeTx I mn∆K was assessed, comparing it with vancomycin, through death curves assay against MRSA USA300. The LyeTx I mn∆K demonstrated high bactericidal efficacy against MRSA cells, with complete reduction in the bacterial load within a short time period. This peptide was able to eliminate a high bacterial load (10^6^ UFC/mL) of MRSA after only 1 h of exposure at concentrations of 5× and 10× the MIC (Figure 1). At a concentration of 2× the MIC, LyeTx I mn∆K was able to completely reduce the bacterial load in just 3 h. In contrast, the bactericidal effect of vancomycin was less potent. After 6 h of incubation, vancomycin produced approximately four-log reduction and required 24 h to completely eliminate MRSA cells. These results highlight the efficient and rapid ability of the LyeTx I mn∆K to kill MRSA.

### 2.3. LyeTx I mn∆K Reduces Preformed Biofilms of MRSA

MRSA has the ability to form biofilms on various surfaces and tissues, including in wounds, and these bacterial structures are particularly problematic because they make the infection chronic and difficult to treat [21]. As LyeTx I mn∆K showed good activity against planktonic MRSA cells, we evaluated whether this compound is also active against mature biofilms formed by this pathogen, which have low susceptibility to currently available antimicrobials. 

**Figure 2:**
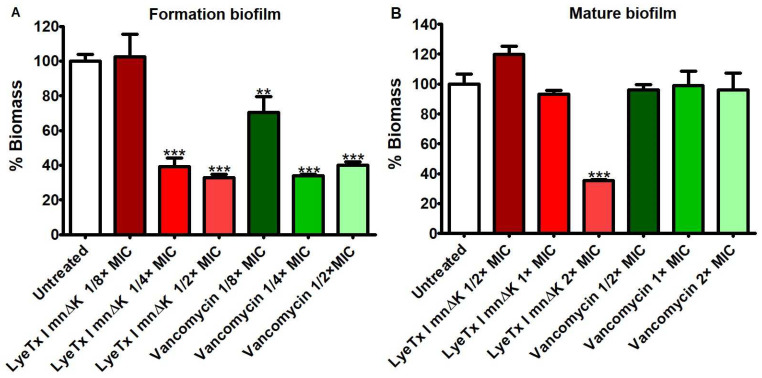
Evaluation of the effect of different concentrations of LyeTx I mnΔK and the control (vancomycin) on the formation of biofilm and pre-formed biofilm of methicillin-resistant *Staphylococcus aureus* (MRSA). (**A**) Reduction in mature biofilm; (**B**) prevention of the formation of biofilm. Two asterisks (**) indicate statistically significant difference compared to the control, with *p*-value < 0.01. Three asterisks (***) indicate statistically significant difference compared to the control, with *p*-value < 0.0001. The results were analyzed by one-way ANOVA with Dunnett’s post-test.

According to the results presented in Figure 2A, the peptide demonstrated a greater ability to reduce biofilm when compared to vancomycin. LyeTx I mnΔK at 2× MIC (16 µM) was able to reduce mature biofilms to 32.28 ± 1.62% of the biomass observed in nontreated cells, while vancomycin did not show any effect, even at the highest concentration tested. Furthermore, we studied the effect of LyeTx I mn∆K and vancomycin on inhibiting MRSA biofilm formation. The peptide reduces the biomass of biofilms formed by MRSA at concentrations of 1/4× MIC (2 µM; 39.28 ± 13.41%) and 1/2× MIC (4 µM; 32.90 ± 5.14%) at a level similar to that observed with the same concentrations of vancomycin (1/4× MIC: 33.93 ± 2.07% and 1/2× MIC: 40.01 ± 5.13%) (Figure 2B). Only at the lowest concentration tested did LyeTx I mn∆K show no significant effect, while vancomycin at 1/8× MIC (0.125 µM) reduces the biofilm formed to 70.43 ± 26.09% when compared to untreated cells (99.91 ± 14.02%). 

### 2.4. Behavior of LyeTx I mnΔK after Combination with Conventional Antimicrobial Agents against MRSA

The combination of the LyeTx I mnΔK peptide with antimicrobials frequently used in the treatment of *S. aureus* infections (i.e., oxacillin and vancomycin) was studied by the synergism and resensitization assay. Initially, the synergism between the peptide and the conventional antimicrobials was evaluated against MRSA using a checkerboard test (Table 2). The combination of vancomycin and LyeTx I mnΔK resulted in a fractional inhibitory concentration index (FICI) of 1.50, indicating an indifferent effect. The combination of peptide with oxacillin showed a FICI of 1.02, which also indicates an indifferent effect. These findings corroborated with the analysis using isobolograms, which showed that the relationship between the FIC of the peptide and the FIC of vancomycin (Figure S1A; Supplementary File) or oxacillin (Figure S1B; Supplementary File) results in a straight line, indicating an additive or indifferent effect.

Next, the MIC values of the antimicrobials (vancomycin and oxacillin) were determined after exposure of MRSA USA300 for 1 h to a subinhibitory concentration of LyeTx I mnΔK. According to Table 3, the results showed that the previous exposition to peptide resulted in a significant increase in the sensitivity of bacteria to the antimicrobials vancomycin and oxacillin. The MIC of vancomycin and oxacillin decreased from 1.0 µg/mL to 0.5 µg/mg and 128 µg/mL to 64 µg/mL, respectively, resulting in a resensitization by two-fold to each antimicrobial agent.

### 2.5. LyeTx I mn∆K Induces Membranolytic Effect on MRSA Cells

**Figure 3:**
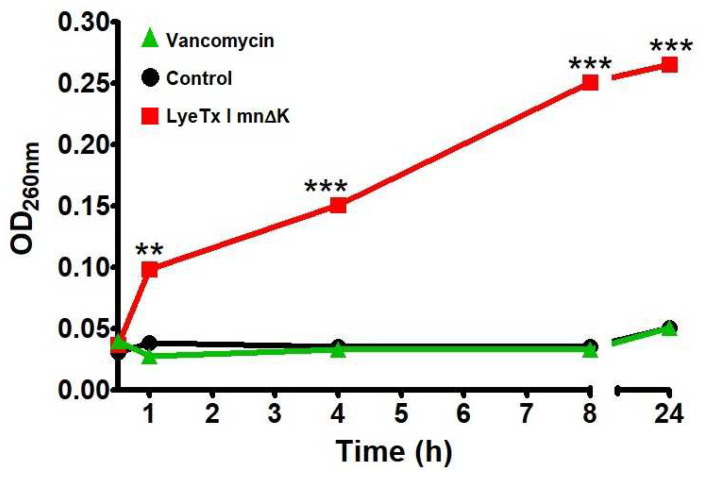
Release of 260 nm-absorbing intracellular material over time for methicillin-resistant *Staphylococcus aureus* (MRSA) suspension treated with LyeTx I mn∆K (80 µM) or vancomycin (10 µM) at 10× MIC. Two asterisks (**) indicate statistically significant difference compared to the control, with *p*-value < 0.01. Three asterisks (***) indicate statistically significant difference compared to the control, with *p*-value < 0.0001. Results were analyzed by one-way ANOVA with Dunnett's post-test.

To assess the ability of LyeTx I mn∆K to damage bacterial membranes the material with absorbance at 260 nm was quantified, suggesting the release of microbial genetic material into the medium after exposure to the compound. In accordance with Figure 3, the treatment of bacterial suspensions of MRSA USA300 with LyeTx I mn∆K at 10× MIC induced a marked time-dependent increase in the 260 nm-absorbance, suggesting that this peptide can disrupt the integrity of the bacterial membrane. In contrast, the activity of vancomycin was similar to the untreated control, indicating that this compound has a low membranolytic effect.

### 2.6. LyeTx I mn∆K Interacts with POPG:CL Membranes

**Figure 4:**
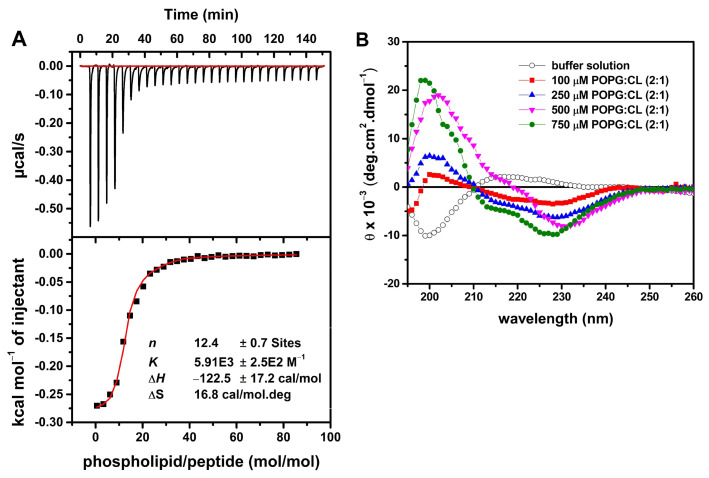
Calorimetric titration isotherms obtained from the titration of a solution of 25 μM LyeTx I mn∆K with 20 mM POPG:CL (2:1, mol/mol) unilamellar phospholipid vesicles (LUVs) (**A**). In the upper panel the heat flow graphs for each LUVs injection are shown as a function of time. The lower panel shows the enthalpy as a function of the phospholipid/peptide molar ratio (the red curve represents the non-linear fitting by eq. (1)). Circular dichroism (CD) spectra of 50 µM LyeTx I mn∆K in the presence of POPG:CL (2:1) (**B**). CD spectra of the peptides in 10 mM Tris, 50 mM NaCl, pH 7.4 are presented by black opened circles, and after addition of different phospholipid concentrations: 100 µM by red filled squares, 250 µM by blue filled up triangles, 500 µM by magenta filled down triangles and 750 µM by filled green circles.

Due to the membranolytic effect observed by the release of intracellular material absorbing at 260 nm, we studied the ability of the LyeTx I mnΔK peptide to interact with the *S. aureus* membrane using techniques that simulate the lipid bilayer of this microorganism. Thus, the membrane–mimetic interactions of *S. aureus* with LyeTx I mn∆K were investigated in aqueous solution and in the presence of membrane–mimetic environments using circular dichroism (CD) spectroscopy. Whereas the CD spectrum of the peptides in aqueous solution is indicative of a predominantly random coil conformation (Figure 4B), the stepwise addition of anionic POPG:CL (2:1) unilamellar phospholipid vesicles (LUVs) to 50 µM of LyeTx I mn∆K suspended in 10 mM Tris, 50 mM NaCl, pH 7.4 results in the appearance of pronounced minima around 208 and 222 nm. 

The interaction between LyeTx I mn∆K and POPG:CL (2:1, mol/mol) LUVs was further investigated by isothermal titration calorimetry (ITC) (Figure 4A). This technique has been extensively used for the characterization of the apparent thermodynamic parameters of the interaction (∆G, ∆H, ∆S, the binding constant K, and the stoichiometric coefficient, n) of some compounds [23,24]. Titration was performed with the peptide solution in the calorimeter cell and the LUVs suspension in the syringe. Figure 4A presents the raw data for the titration of a 25 µM solution of LyeTx I mn∆K with 20 µM POPG:CL LUVs. The first injections of LUVs into the peptide solution shows an exothermic heat (~−55 µcal/s) associated with the peptide–membrane interaction, which reaches a plateau when a LUVs/peptide molar ratio of 20 is reached. The enthalpy calculated for each injection of LUVs subtracted from the enthalpy of LUVs dilution is plotted as a function of the phospholipid/peptide ratio in Figure 4A. 

The inflection point of the isotherm corresponds to the stoichiometric molar ratio (n). The n of approximately 12 indicates a supramolecular peptide–LUVs structure containing 12 lipids for each peptide molecule of the system under analysis. The binding isotherm was calculated by non-linear curve fitting using the model of equivalent binding sites (Equation (1)) and the thermodynamic parameters for the peptide–membrane interaction are shown in Figure 4A. The peptide–membrane binding is driven by both enthalpic and entropic contributions, where the entropic term (588 cal/mol) is around five times greater than the enthalpic contribution (−122 cal/mol).

### 2.7. LyeTx I mn∆K Has Low Interaction with Artificial Vesicles That Mimic Eukaryotic Membranes 

**Figure 5:**
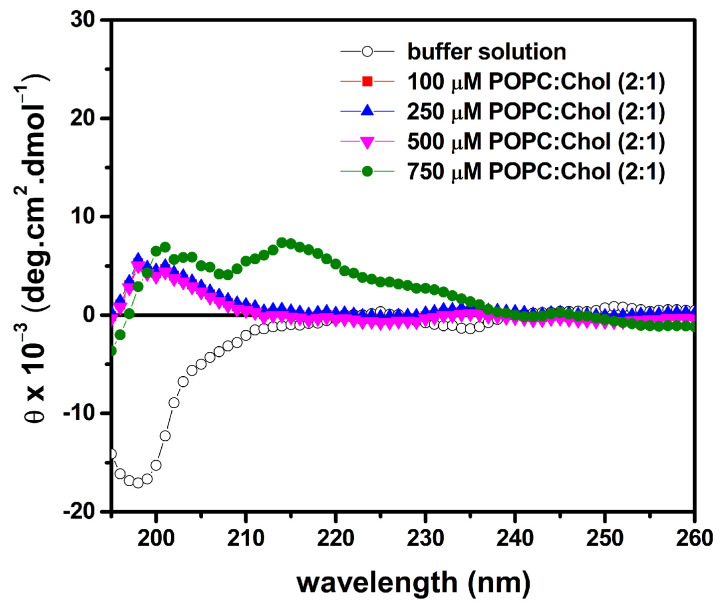
Circular dichroism (CD) spectra of 50 µM LyeTx I mn∆K in the presence of POPC:Chol (2:1). The CD spectra of the peptide in 10 mM Tris, 50 mM NaCl, pH 7.4 are presented by black open circles, and after addition of different phospholipid concentrations: 100 µM by red filled squares, 250 µM by blue filled up triangles, 500 µM by magenta filled down triangles and 750 µM by filled green circles.

The conformational behavior and lytic activity of LyeTx I mn∆K in the presence of mimetic eukaryotic membranes were investigated by CD and calcein release, respectively. The LyeTx I mn∆K spectra reveal any defined minimal in the presence of zwitterionic POPC LUVs containing 33% cholesterol (Chol) mol/mol (Figure 5).

**Figure 6:**
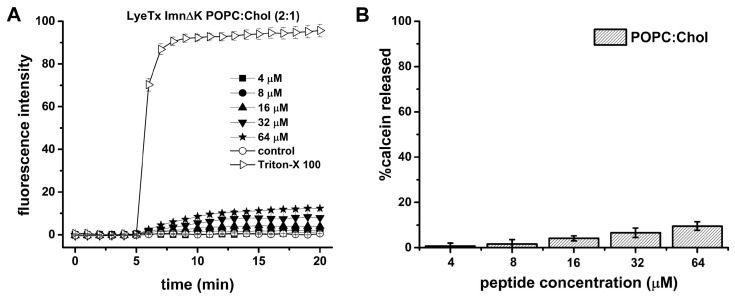
Effect of LyeTx I mn∆K on phospholipid membranes as assessed by calcein release from POPC:Chol (2:1) at 100 µM lipid concentration. (**A**) Time dependence of membrane permeability with LyeTx I mn∆K. (**B**) Percentage of calcein release as a function of peptide concentration (positive control—2% Triton X-100, considered as 100%). Data are representative of three independent experiments.

The ability of the peptide to induce leakage from calcein-loaded LUVs made of POPC:Chol (2:1) was evaluated in the presence of 100 µM lipid concentration. The calcein-loaded LUVs with the addition of the peptides are presented in Figure 6A,B. 

### 2.8. Effect of LysylPOPG on the Peptide–Membrane Interaction 

**Figure 7:**
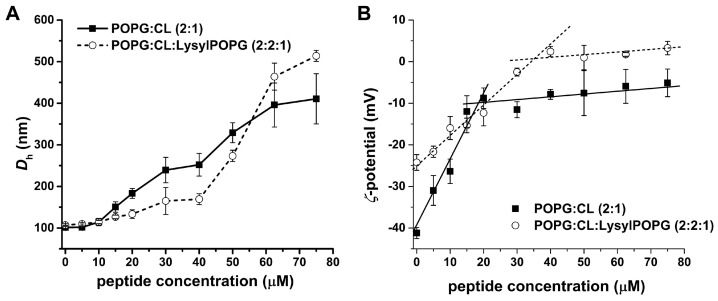
Effect of LyeTx I mn∆K on the size and charge of 200 µM POPG:CL (2:1 mol/mol) and 200 µM POPG:CL:LysylPOPG LUVs (2:2:1 mol/mol/mol). (**A**) Changes in the hydrodynamic diameter (D*_h_*) and (**B**) in the zeta potential as a function of the peptide concentration. Data are representative of three independent experiments.

In order to show the effect of the membrane–mimetic interactions of *S. aureus* with peptide upon the hydrodynamic diameter (*D_h_*) and the zeta potential (*ζ*P) of POPG:CL (2:1) and POPG:CL:LysylPOPG (2:2:1) LUVs, a time lapse dynamic light scattering (DLS) experiment was performed (Figure 7) [25,26,27]. The DLS experiments were carried out by incubating the respective LUVs suspension in the absence and presence of 5–75 μM LyeTx I mn∆K for 30 min. Figure 7A shows the changes in the D*_h_* of vesicles upon addition of the peptide. The control vesicles were monodisperse with a D*_h_* of 101 ± 5 nm, while for POPG:CL:LysylPOPG a *D_h_* of 107 ± 4 nm was observed. Both LUVs incubated with peptide showed an increased hydrodynamic diameter in a concentration-dependent manner. The D*_h_* slightly increase until 10 µM of peptide concentration, with a ∆*D_h_* of 13 nm for the LUVs in absence of LysylPOPG and 7 nm for the LUVs containing LysylPOPG. Above this concentration, the D*_h_* values ramp up with addition of the peptide in both lipid systems, reaching around four and five times higher D*_h_* than those observed for the control vesicles (410 ± 60 nm for POPG:CL and 515 ± 15 nm for POPG:CL:LysylPOPG). 

The electrophoretic mobility of LUVs in the presence of LyeTx I mn∆K was measured to evaluate the effect of the peptide on the zeta potential (ζP) of both lipid systems. Figure 7B presents the ζ-potential experiments in which LUV suspensions were titrated with solutions of LyeTx I mn∆K. The initial values of ζP are near −41 mV and −26 mV for POPG:CL and POPG:CL:LysylPOPG LUVs, respectively. The addition of peptide led to a continuous increase in the ζP of the anionic vesicles until a peptide concentration of 20 µM in the presence of POPG:CL and 40 µM in the presence of POPG:CL:LysylPOPG LUVs. From these values, a plateau is reached with a change of ζP around −30 mV and −25 mV in the presence of the POPG:CL and POPG:CL:LysylPOPG LUVs, respectively.

**Figure 8:**
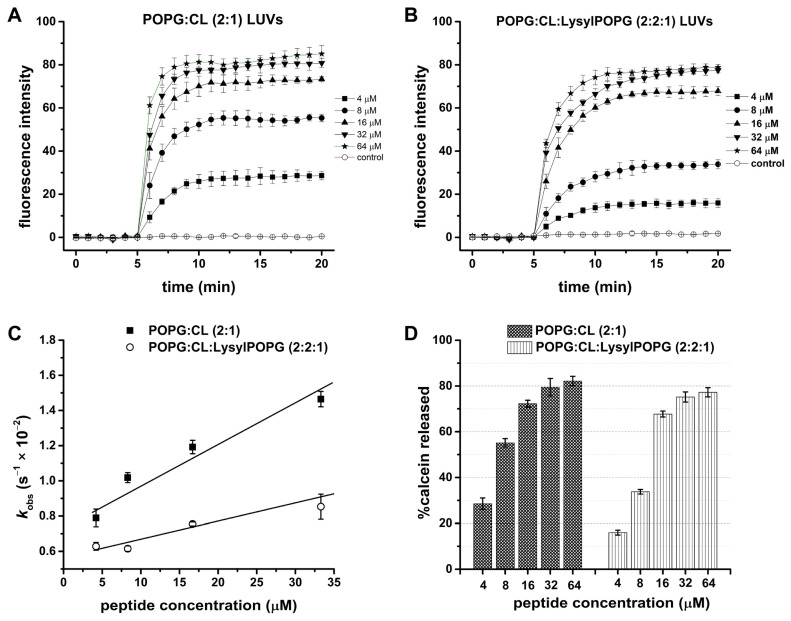
Effect of [LyeTx I mn∆K] on phospholipid membranes as assessed by calcein release from POPG:CL (2:1) and POPG:CL:LysylPOPG LUVs (2:2:1), both at 100 µM of lipid concentration. Time dependence of membrane permeability with LyeTx I mn∆K (**A**) POPG:CL (2:1); (**B**) POPG:CL:LysylPOPG LUVs (2:2:1); (**C**) observed rate constants, k_obs_, of CF release vs. peptide concentration, and (**D**) percentage of calcein release compared to what was observed after addition of 2% Triton X-100 (positive control, considered as 100%). Data are representative of three independent experiments.

The ability of LyeTx I mn∆K to induce leakage on calcein-loaded LUVs was studied on POPG:CL (2:1) or POPG:CL:LysylPOPG (2:2:1), both at 100 µM of lipid concentration. The results of the leakage measurements of the calcein-loaded LUV with the addition of LyeTx I mn∆K at 35 °C are presented in Figure 8. The calcein leakage from both LUVs is virtually negligible in the absence of the peptide (first 5 min). Calcein release is immediately observed after the LyeTx I mn∆K is added (Figure 8A,B). Kinetics of the calcein release were recorded for 15 min and the maximum effect of the peptide on both LUVs is observed around after 6 min in POPG:CL and 9 min in POPG:CL:LysylPOPG, after which the disrupting effect was stable and did not increase with time. The addition of the peptide in increasing concentrations to the dye-containing LUV induced calcein release in both systems in a concentration-dependent manner. The rate of calcein leakage from the vesicles was calculated by the increase in fluorescence emission as a function of time. The data were analyzed as a first-order process and a rate constant, K_obs_, was calculated for each run. 

The kinetics of calcein release from POPG:CL and POPG:CL:LysylPOPG LUVs are presented in Figure 8C and the rate constants are shown in Table 4. For both LUVs, increments in the LyeTx I mn∆K concentration led to a linear increase in the rate of calcein leakage (k_obs_). The slope for the plot of the k_obs_ vs. peptide concentration for POPG:CL:LysylPOPG LUVs is slightly lower than the value obtained for POPG:CL LUVs, indicating a lower kinetics of calcein release. The percentage of calcein release (% calcein release), after 15 min, was determined by using Equation (2) (see Section 4.10) and the values were plotted as a function of peptide concentration (Figure 8D) in the presence of either POPG:CL or POPG:CL:LysylPOPG lipid systems. In the presence of 4 µM LyeTx I mn∆K the disrupting effect was higher for POPG:CL LUVs (28.5% ± 2.5) when compared to 16.0 ± 1.0% for POPG:CL:LysylPOPG. Nevertheless, the disrupting effect of the peptide was similar in both vesicles at the highest (32 µM) LyeTx I mn∆K molar concentration assayed; the leakage was 82.2 ± 3.0% for POPG:CL and 77.2 ± 2.0% for POPG:CL:LysylPOPG vesicles.

In order to evaluate the lytic activity of LyeTx I mn∆K in the eukaryotic membranes, similar leakage experiments were also carried out in the presence of calcein-loaded LUVs composed by POPC:Chol (2:1) (Figure S2; Supplementary File). Notably, the calcein leakage from zwitterionic LUVs is at least eight times lower than the values observed in the presence of both anionic LUVs. Whereas the calcein leakage from POPG LUVs is around 80%, the maximum value reached in the presence of POPC:Chol LUVs is less than 10%.

### 2.9. Gel Containing LyeTx I mnΔK Is Effective in MRSA-Induced Wounds in Mice

**Figure 9:**
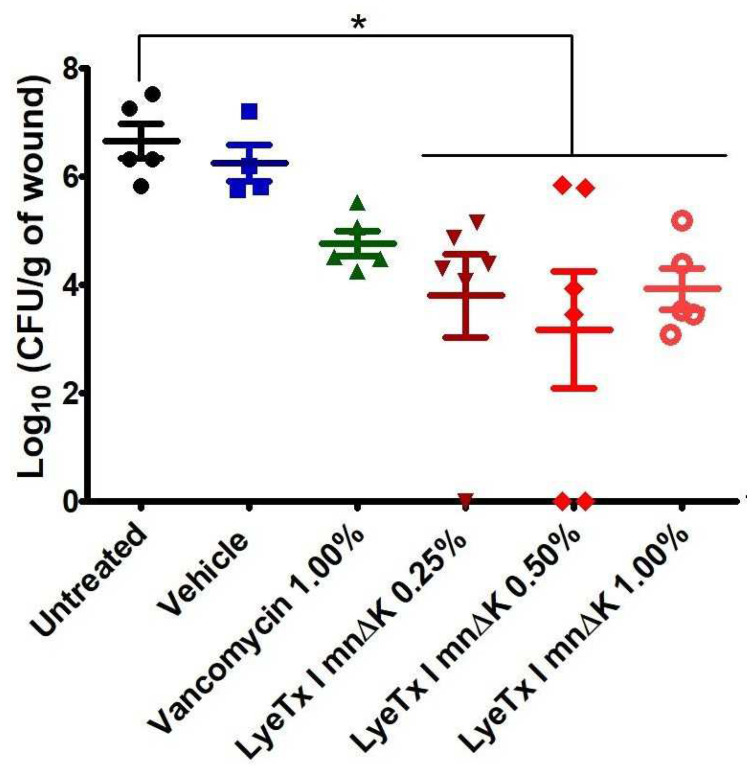
Bacterial load (Log_10_CFU/g of wound) after the topical treatment of non-surgical methicillin-resistant *Staphylococcus aureus* (MRSA)-infected wounds with gel containing LyeTx I mnΔK (0.25%, 0.50%, or 1.00%), vancomycin 1%, gel ointment, or saline (control) (*n* = 5). Mice were intradermally injected with 10^7^ CFU of highly virulent MRSA USA300. Approximately 48 h after injection, the mice developed an open wound/abscess at the local site of injection and were treated twice daily for 3 days. An asterisk (*) indicates a statistically significant difference compared to the control with *p*-value < 0.05. All results were analyzed by one-way ANOVA with Dunnett’s post-test.

Due to the relevant antibacterial activity of the LyeTx I mnΔK peptide in vitro, we evaluated the use of a gel formulated with this active compound in the treatment of wounds induced by MRSA in vivo. Thus, to assess the potential of LyeTx I mnΔK as a topical pharmacological agent, mice were infected with *S. aureus* MRSA USA 300 by intradermal injection, allowing the formation of an open wound/abscess. After treatment, a significant reduction in the bacterial load from lesions was observed in mice exposed to LyeTx I mnΔK when compared to the untreated groups (6.66 ± 0.71 Log_10_ CFU/g of wound) or those treated with base gel (6.25 ± 0.67 Log_10_ CFU/g of wound) (*p*-value < 0.05; Figure 9). Mice treated with gel containing 0.50% LyeTx I mnΔK showed the greatest reduction in bacterial load (3.18 ± 2.64 Log_10_ CFU/g of wound), followed by those that received 0.25% LyeTx I mnΔK (3.81 ± 1.90 Log_10_ CFU/g of wound) and 1.00% LyeTx I mnΔK (3.93 ± 0.85 Log_10_ CFU/g of wound) (Figure 9). Therefore, the effect of the peptide in vivo did not show dose-dependent behavior. In turn, the gel containing 1% vancomycin only showed a small reduction in the bacterial load of the wound (4.78 ± 0.52 Log_10_ CFU/g of wound) when compared to the untreated group, revealing that the treatment with LyeTx I mnΔK, even at the lowest concentration tested (0.25%), was more effective than the vancomycin. These results corroborate the finding that LyeTx I mnΔK is a promising molecule for topical administration and is, thus, superior to reference drugs.

**Figure 10:**
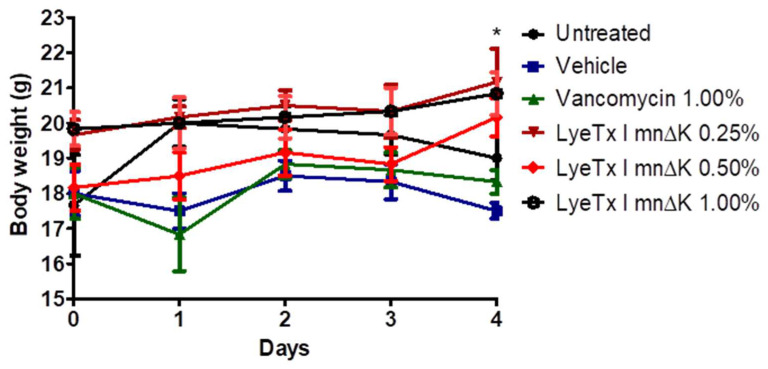
Effect of the peptide on changes in body weight in mice infected with methicillin-resistant *Staphylococcus aureus* (MRSA). An asterisk (*) indicates a statistically significant difference between LyeTx I mnΔK 0.50% and untreated group with *p*-value < 0.05. All results were analyzed by one-way ANOVA with Dunnett's post-test.

Corroborating these findings, the animals that received the gel containing the peptide at 0.50% showed a significantly lower weight loss compared to untreated animals (Figure 10). This result suggests that the discomfort of the wound, which leads to less water and food intake with consequent weight loss in animals, was reduced after using the formulation containing the peptide.

## 3. Discussion

Several studies indicate that the spread of infections caused by MRSA is a major public health challenge worldwide [28]. In this sense, the World Health Organization (WHO) categorized this superbug as a high priority microorganism for the search and development of new antimicrobials [28], and AMPs stand out as relevant agents in this scenario. Here, we showed that a synthetic peptide inspired by a compound isolated from the venom of the *L. erythrognatha* spider, called LyeTx I mnΔK, has good activity against planktonic cells and biofilms of MRSA, in addition to being useful in the treatment of wounds caused by this superbug [29].

The peptide LyeTx I mnΔK showed remarkable antibacterial activity against the MRSA isolates tested, having a pronounced bactericidal action at low concentrations and with rapid maximum effect. In the treatment of infections, it is important that the antimicrobial agent acts quickly and can completely eradicate the microorganism to prevent the development of microbial resistance and complications of infection [19,21]. In this regard, the predominantly bactericidal activity of peptide has shown promise, especially in highly resistant microorganisms such as MRSA. In contrast to the rapid bactericidal effect of LyeTx I mnΔK, vancomycin exhibits a very slow antibacterial activity, which may compromise its efficacy. Clinical use of vancomycin often leads to therapeutic failure due to its limited bactericidal capacity [30], which makes LyeTx I mnΔK advantageous because of its efficient microbicidal property.

In addition to its potential to acquire antimicrobial resistance genes, *S. aureus* exhibits another virulence factor in its ability to form biofilms. Bacterial biofilms are clusters of bacteria that are attached to a surface and/or to each other and are embedded in a self-produced exopolymer matrix that protects them from adverse conditions such as sanitizers and antimicrobials agents [31]. When MRSA-formed biofilms are established in wounds, they exhibit resistance to destruction by conventional antimicrobials, which involves a high risk of inflammation and/or purulence [32]. Therefore, the activity of the LyeTx I mnΔK peptide was evaluated against MRSA biofilms and the results revealed that this compound has a promising anti-biofilm effect. These results were similar to those observed by Lima et al. (2021) who showed that LyeTx I mnΔK has the ability to disrupt mature biofilms and inhibit the formation of this bacterial structure in carbapenem-resistant *Acinetobacter baumannii* cells [20]. It is worth noting that the effect of the peptide on mature biofilms formed by MRSA was superior to the observed effect of vancomycin. Conventional antimicrobials have a low ability to act on biofilms because, in addition to the exopolymeric matrix that physically protects bacterial cells from interaction with these compounds, the microorganisms within the biofilm are in a stationary phase of growth [33]. As all available antibacterial agents only exhibit activity against cells in the logarithmic growth phase, bacteria within the biofilm are naturally insensitive to the action of these drugs [34,35]. Therefore, the present result shows that the LyeTx I mnΔK peptide, different from vancomycin, can act in both phases of microbial growth, i.e., logarithmic and stationary.

A rapid bactericidal effect and activity on bacterial biofilms has been associated numerous times with a compound’s ability to damage the bacterial cell membrane, which can result in lysis. In fact, the LyeTx I mnΔK peptide significantly increases the loss of genetic material from MRSA cells, as indicated by the release of 260-nm-absorbing intracellular compounds, showing that this AMP damages the bacterial plasma membrane. This characteristic of AMPs to cause membrane damage, associated with a rapid and instantaneous bactericidal action, has been highlighted by several authors such as Brogden (2005) [10] and Avelar (2015) [36]. In addition to being associated with a rapid antimicrobial effect, the action of LyeTx I mnΔK on the MRSA membrane may come with other advantages such as the reduced potential for inducing resistance and low dosage requirements in clinical use. In this context, it is widely known that compounds that act on plasma membrane have a low capacity to induce antimicrobial resistance because membrane redesign by bacteria would be a “costly” solution for most microbial species [37,38]. Moreover, due to the low MIC values related to these antimicrobial agents, the concentrations normally required for clinical use are low, which reduces the possibility of adverse events related to this medication [9]. Vancomycin, in turn, did not alter the release of materials with absorption at 260 nm, which suggests a discrete effect on the MRSA membrane. This glycopeptide acts by binding to the end of the cross-links that form the bacterial wall (specifically, to the D-Ala-D-Ala residues) [39], therefore, it does not interfere with the bacterial lipid membrane, which is confirmed by our results.

Despite the numerous advantages that the studied compound presents over vancomycin, alpha-helical AMPs such as LyeTx I mnΔK usually show disadvantages that may limit their clinical use. These agents have recognized toxicity, especially renal and cerebral, are quickly eliminated by glomerular filtration due to low binding to plasma proteins, have a large volume of distribution, are very sensitive to degradation by serum peptidases, and are compounds that have a high production cost [15,29,40]. Therefore, in order to avoid several of these limitations that could make the therapeutic use of LyeTx I mnΔK unfeasible, we chose to develop a topical formulation containing the compound. In fact, numerous studies have revealed that its use as a topical pharmacological agent is dependent on overcoming several of the intrinsic disadvantages of AMPs [41,42]. Furthermore, our research group has already been working on chemical modifications (e.g., PEGuilation, glycosylation, and the addition of halogen atoms at aromatic residues) to this peptide seeking to reduce their toxicity, increase plasma half-life, and protect against hydrolysis by circulating proteases.

In order to investigate the effect of LyeTx I mn∆K on MRSA membranes, we studied the peptide–membrane interaction in the presence of POPG:CL (2:1, mol/mol). This phospholipid composition was selected as it represents the predominant constituents of *S. aureus* membranes [43,44,45]. Initially, the thermodynamic studies regarding the membrane interactions of LyeTx ImnDK with POPG:CL LUVs indicated that the peptide presents a moderate to high affinity with negatively charged vesicles at 35 °C. Although, the negative enthalpy values indicate exothermic binding of the peptides to the anionic vesicles and the predominance of attractive Coulomb forces, the binding process for the peptide is mainly driven by entropic factors, since |TΔS| >> |ΔH|. This interaction should occur by partial peptide incorporation in the bilayers interface, as has been shown by the increase in vesicle size observed by the DLS experiments [46]. Interestingly, above this critical concentration, the PDI values ramp up (>0.3) and two size populations were noted. This suggests that the peptide adsorption to the membranes is followed by vesicle aggregation at high peptide concentration (>20 μM) [22,47]. Notably, the peptide concentration dependence is also observed in the ζ-potential measurements. Initially, the interaction of the positively charged peptide with the negatively charged bilayers results in a linear increase in DP, reaching a plateau between 15 and 20 μM peptide.

Regarding the secondary structure of the peptide, our results show *α*-helical structures when in contact with membrane surface of POPG:CL [48]. LyeTx I mn∆K presents a significant affinity to both phospholipid vesicles, with an association constant (5.9 × 10^3^ M^−1^) that falls within the range of 10^3^–10^4^ M^−1^ [46,49]. However, in the presence of POPC:Chol (2:1) lipid bilayers, mimicking eukaryotic membranes, the LyeTx I mn∆K spectra reveal no defined conformation. These results suggest that the shortened form presents a lower affinity to the eukaryotic mimetics membranes. The same can be observed with the calcein release, in which the LyeTx I mn∆K reached a level of less than 20% in vesicles that simulate mammalian cells compared to membranes related to the envelope of *S. aureus*. Corroborating these findings, previous studies have shown that the LyeTx I mnΔK peptide has reduced cytotoxicity activity against eukaryotic cells. The concentration required to kill 50% (CC_50_) of Lund human mesencephalic cells (LUHMES; CC_50_ 15.02 µM) [18] and African green monkey (*Chlorocebus aethiops*) kidney epithelium cells (Vero; CC_50_ 55.31 ± 5.0 µM) [20] is 1.8 at 6.6 times higher than the MIC_50_ observed against MRSA clinical isolates in this study. Furthermore, LyeTx I mnΔK has a hemolytic concentration higher that its antimicrobial dose, with a cytotoxic concentration for 50% of mammalian erythrocytes ranging from 44.67 µM to 81.17 µM [18,20]. 

Lysyl phosphatidylglycerol (LysylPOPG) is commonly found in the multidrug-resistant *Staphylococcus aureus* such as MRSA by modification of POPG. This process is driven by multiple peptide resistance factors (MprF), which modify the phospholipids with positively charged lysine residues in the cytoplasmic membrane leaflet and translocate the LysylPOPG to the outer cytoplasmic membrane [50]. This modification has shown resistance to cationic antimicrobial peptides such as colistin, nisin, human-β-defensin 3, and polymyxin B. 

In order to explore the effect of the addition of LysylPOPG lipid in membrane permeabilization ability of the LyeTx I mn∆K, we evaluate the D*_h_*, ζP and calcein release from LUVs containing LysylPOPG. Although the addition of LysylPOPG decreases the kinetics (k_obs_) of lytic activity for LyeTx I mn∆K, this lipid does not affect the maximum release of calcein caused by the peptide. The effect of the peptide was also virtually equivalent to the changes of ζP of the LUVs in the absence and in the presence of LysylPOPG (*ζ*P ~ −30 mV for POPG:CL and −25 mV for POPG:CL:LysylPOPG). The polydispersity index (PDI) of the suspensions increased, suggesting that peptide–membrane interaction affects the originally homogeneous vesicles resulting in a deformed lipid–peptide complex. Interestingly, two populations can be observed with increasing of peptide concentration, one centered around 100 nm and the other around 1000 nm (Figure S3; Supplementary File). These findings suggest a fusion process as compared to aggregation which results in the polydispersity of the size population [51,52]. Similarly, the effect of the peptide in the D_h_ of both LUVs was comparable, resulting in vesicle agglutination. These findings are in accordance with the antimicrobial assays, which have demonstrated high activity of LyeTx I mn∆K, even against the MRSA USA300 strains (MIC_50_ and CBM_50_ of 8 µM and 32 µM, respectively). Thus, the presence of LysylPOPG, which constitutes part of the adaptation of MRSA to antibiotic treatments, is surpassed by LyeTx I mn∆K, indicating its effectiveness even in challenging conditions. 

One of the most commonly used strategies for treating infections caused by MDR bacteria, such as MRSA, is the combination of different antimicrobial agents [53]. This therapeutic protocol has several advantages such as rapid antibacterial effect, lower possibility of therapeutic failure due to selection of resistant bacteria, reduced concentration of each antimicrobial used in the combination, and lower risk of serious adverse reactions associated with high doses of the drug [54]. LyeTx I mn∆K is indifferent to the activity of the antimicrobials oxacillin and vancomycin. This indicates that both compounds can be combined to broaden the spectrum of antimicrobial action, thus helping to treat polymicrobial infections [38]. Furthermore, prior exposure to LyeTx I mn∆K reduces the active concentrations of these two antimicrobials, which may be beneficial in reducing the dose of these agents with a consequent lower frequency of adverse reactions [54]. 

Clinical manifestations of MRSA range from auto-limited superficial infection to life-challenging bacteremia; however, wounds are still the most common problem caused by this pathogen [55]. MRSA-infected wounds are difficult to heal, purulent, and painful. In addition, the treatment of this dermatological infection is restricted to a few options such as vancomycin, mupirocin, and clindamycin [56]. Here, we show that a formulated gel containing LyeTx I mnΔK was effective in treating MRSA-induced non-surgical wounds in mice, reducing bacterial load in a non-dose-dependent manner. Interestingly, this effect was superior to that of the reference drug (vancomycin). Due to their amphoteric nature, AMPs have the ability to better interact with the corneal extract of the epidermis. This characteristic facilitates absorption through the skin, allowing them to reach deeper layers of the dermis, including infection sites. Thus, the LyeTx I mnΔK peptide is highlighted as a promising alternative for the treatment of cutaneous infections by MRSA. On the other hand, vancomycin, being a hydrophilic molecule [57], faces limitations in penetrating through the skin. This characteristic hinders its access to deeper layers of the dermis, compromising its effectiveness in treating deep skin infections.

## 4. Materials and Methods

### 4.1. Microorganisms

The antibacterial activity was evaluated against six clinical isolates of methicillin-resistant *Staphylococcus aureus* (MRSA). All isolates were recovered from wounds of patients with acute infections admitted to Santa Casa de Belo Horizonte. The procedure was approved by the ethics committee for human subjects of Group Santa Casa BH (CAAE: 09187719.4.0000.5138). Bacterial identification was performed by automated biochemical-physiological methods (BD Phoenix^®^; Frankfurt, Germany) and MRSA phenotype was confirmed by disk-diffusion assay using cefoxitin [58]. The reference strain *S. aureus* MRSA USA300 was kindly provided by the Reference Laboratory in Microbiology at the Oswaldo Cruz Foundation (FIOCRUZ-RJ, Rio de Janeiro, Brazil).

### 4.2. Reagents

Vancomycin (Inlab, São Paulo, SP, Brazil), dimethyl sulphoxide (DMSO), sodium chloride (NaCl), ethylic alcohol, glacial acetic acid, violet crystal (Synth, São Paulo, SP, Brazil), and oxacillin (Sigma-Aldrich, San Louis, MO, USA) were all purchased from commercial suppliers. The peptide LyeTx mnΔK was obtained by solid phase synthesis and acquired from GL Biochem (Shanghai, China) with a purity of 99%, which was confirmed by spectrometric analysis realized by the chemistry department of *Universidade Federal dos Vales do Jequitinhonha e Mucuri (UFVJM)*. Mueller–Hinton broth, Mueller–Hinton agar, Mannitol agar, and trypticase soy broth (TSB) were purchased from Kasvi (São José do Pinhais, PR, Brazil).

### 4.3. Antimicrobial Activity

#### 4.3.1. Preparation of the Inoculum

The bacterial inoculum used in the susceptibility test was standardized according to the Clinical Laboratory and Standard Institute (CLSI) M07 document (CLSI, 2018). Initially, bacterial streaks were performed on Mueller–Hinton agar. Subsequently, three to five isolated colonies from the *S. aureus* cultures were selected and transferred to tubes containing sterile saline solution (0.9% NaCl). The resulting suspension was adjusted to the 0.5 McFarland scale (corresponding to 10^8^ CFU/mL) using a spectrophotometer (Shimadzu UVmini-1240 Spectrophotometer UV/Vis Reader). Finally, 50 μL of this suspension was transferred to 10 mL of Mueller–Hinton broth, resulting in a final inoculum at 10^6^ CFU/mL, which was employed in antimicrobial tests.

#### 4.3.2. Determination of Minimum Inhibitory Concentration (MIC) 

Mueller–Hinton broth [20] was used for determination of minimum inhibitory concentration (MIC) by broth microdilution method, according to the CLSI document M07 for aerobically growing bacteria. The compounds (LyeTx I mnΔK and vancomycin) were dissolved in sterile water and serially diluted two-fold, ranging from 0.5 to 128 µM. Finally, the results were visualized after 24 h of incubation at 37 °C and the MIC value was considered as the lowest concentration of tested compound able to prevent visible bacterial growth. Vancomycin and wells with inoculum and culture medium were included as positive and negative controls, respectively. Furthermore, wells exclusively containing culture medium were included as a control for medium sterility, and all experiments were performed in triplicate.

#### 4.3.3. Determination of Minimum Bactericidal Concentration (MBC)

The minimum bactericidal concentration (MBC) was determined by transferring 100 µL from the wells optically free of growth of MIC assay to plates of Mueller–Hinton agar plates. After 24 h of incubation at 37 °C, the MBC was considered as the lowest concentration of the tested agent capable of reducing 99% of colonies in relation to the negative control. Vancomycin was used as positive control.

### 4.4. Time-Kill Curve

The peptide activity as a function of time was studied by death curve assay. Initially, an inoculum of MRSA USA300 at 10^6^ UFC/mL was prepared, conforming to Section 4.3.1. Bacteria were then challenged with LyeTx I mn∆K at the 2×, 5×, and 10× MIC. Untreated cells and vancomycin at 10× MIC were used as negative and positive control, respectively. The tubes were then incubated at 37 °C with aeration at 225 rpm. At intervals of 0, 30, 60, 120, 150 and 180 min, 100 μL of this bacterial suspension was serially diluted (10^−1^ to 10^−5^) in sterile saline (0.9% NaCl) and plated onto mannitol agar. The plates were finally incubated for 24 h and the CFU/mL of morphotypes colonies of *S. aureus* on mannitol agar (i.e., lysis, medium, and yellow colonies) was calculated [31].

### 4.5. Anti-Biofilm Activity

To assess the effect of the LyeTx I mn∆K on pre-formed biofilm of MRSA USA300, as well as on the formation of biofilm by this bacterium, the methodology described by Herrera et al. (2020) was utilized [59]. Mueller–Hinton broth supplemented with glucose 1% (MHBg) and a bacterial suspension prepared according to Section 4.3.1 were used. In the biofilm formation inhibition assay, a bacterial suspension in MHBg was incubated at 37 °C for 24 h with the compounds (LyeTx I mnΔK and vancomycin) at concentrations below the MIC (1/8×, ¼×, 1/2× MIC), and inoculum without treatment was considered a control. Next, the medium was removed, and the microtiter plates washed three times with saline (NaCl 0.9% *w*/*v*). In the established biofilm disruption assay, bacterial suspension in MHBg was incubated at 37 °C for 24 h to enable biofilm formation. After removal of the medium, the microtiter plates were washed three times with saline and the biofilm attached to the wells treated with the compounds (LyeTx I mnΔK and vancomycin) at 1×, 2× and 4× MIC. Bacterial suspension without treatment was considered a control (untreated group). The microtiter plates were incubated at 37 °C for 24 h, the medium was removed and then washed three times with saline.

In both assays, the biofilm was fixed in wells with 100 µL methanol for 30 min at 37 °C. Next, the wells were washed with saline (three times) and biofilms were stained with 1% *w/v* crystal violet for 30 min at room temperature. Finally, the wells were washed with saline (three times), air-dried for 2 h, and the biofilm masses solubilized in 30% glacial acetic acid. The readings were taken on a microplate reader (Bio-Tek Instruments, Winooski, VT, USA) at 600 nm. The biofilm biomass percentages in treated groups were calculated by comparing their absorbance values with those of untreated wells (100% of biomass). Results of biofilm assays were presented graphically using the arithmetic mean ± standard deviation of six independent experiments. 

### 4.6. Membranolytic Effect

In this study, the effect of compounds (LyeTx I mn∆K and vancomycin) on membrane of MRSA USA300 was evaluated through of the release of intracellular material with absorbance at 260 nm [38]. Tubes containing 5 mL of a suspension of MRSA USA 300 at 10^8^ CFU/mL in saline, prepared according to item 3.4.1, were exposed to the LyeTx I mn∆K or vancomycin at 10× the MIC of each one. After incubation at 37 °C, a 500 µL sample of tubes was removed at different time intervals (0, 0.5, 1, 3, 6 and 24 h), centrifuged (1500× *g* for 25 min at 4 °C) and the supernatant removed to measure the optical density at 260 nm in a spectrophotometer (Bio-Tek Instruments, Winooski, VT, USA). Untreated cells were included as controls and the results were expressed graphically as OD_260nm_ vs. time (hours). 

### 4.7. Combination Assays 

#### 4.7.1. Synergism

The interaction of LyeTx I mnΔK with vancomycin and oxacillin against MRSA USA300 was evaluated by the checkerboard assay as described previously [60]. *S. aureus* suspensions (10^6^ CFU/mL) were incubated with the combination of LyeTx I mnΔK and antimicrobials (0.05–40 μg/mL) at ratio of 1:1 for 24 h at 37 °C. Next, the fractional inhibitory concentration index (FICI) was calculated as the sum of the FICs of the peptide and the FIC of the antimicrobials, which are defined by the following equations:

(1) $$FIC\;(LyeTx\;I\;mn\Delta K)=\frac{MIC\;LyeTx\;I\;mn\Delta K\;combined}{MIC\;LyeTx\;I\;mn\Delta K\;alone}$$

(2) $$FIC\;(antimicrobial)=\frac{MIC\;antimicrobial\;combined}{MIC\;antimicrobial\;alone}$$

The results were interpreted according to Oroojalian et al. (2002) [61], which considered the effect synergic if FICI ≤ 0.5, additive if 0.5 < FICI ≤ 1.0, indifferent if 1.0 < FICI ≤ 4, and antagonist if FICI > 4.0. Additionally, the results were interpreted by plotting the FIC values of each compound involved in the combinations. Thus, a graph called isobologram is generated, where agents are considered synergistic when the orthogonal projection is concave, additive or indifferent if the projection behaves like a straight line, and antagonistic in cases where the projection takes a convex form [38].

#### 4.7.2. Resensitization

The effect of previous exposure of MRSA USA300 to LyeTx I mnΔK on MIC values of antimicrobial agents (oxacillin and vancomycin) were performed as described previously [62], with modifications. Briefly, 1/4× MIC of LyeTx I mnΔK was incubated with the bacterial suspension (10^6^ CFU/mL) in MHB at room temperature for 60 min. After incubation, the tube was centrifugated at 1500× *g* for 25 min at 4 °C, the supernatant was removed and 10 mL of sterilized MHB was added to the tube. Next, peptide-treated bacteria were added to 96-well plates and the MIC of oxacillin and vancomycin was determined. Peptide-untreated bacteria served as negative control. The result was expressed as the fold-change of resensitization, calculated by the ratio of the MIC of antimicrobial alone and the MIC of the antimicrobial after resensitization with 1/4× MIC of LyeTx I mnΔK.

### 4.8. Preparation of Large Unilamellar Vesicles (LUVS)

LUVs were prepared using the phospholipids, 1-palmitoyl-2-oleoyl-*sn*-glycero-3-phospho-(1′-rac-glycerol) (POPG), diphosphatidylglycerol (Cardiolipin—CL), and 1,2-dioleoyl-sn-glycero-3-[phospho-rac-(3-lysyl(1-glycerol)) (LysylPOPG). The appropriate amount of phospholipid in a molar ratio of POPG:CL 2:1 or POPG:CL:LysylPOPG 2:2:1 was transferred to a glass tube and suspended with 1 mg per mL of chloroform at room temperature. The organic solvent was removed in a rotary evaporator to form a lipid film. Multilamellar vesicles (MLVs) were prepared according to the freeze thawing method in aqueous buffer (10 mM Tris-HCl, 50 mM NaCl, pH 7.4), eventually containing calcein (50 mM) at 25 °C. The calcein buffer was prepared by diluting 545 mg of calcein in 2.5 mL of 1 M NaOH, followed by addition of 1.25 mL of buffer (100 mM Tris-HCl, 50 mM NaCl, pH 7.4), together with 2.5 mL of Milli-Q water (Millipore, Bedford, MA, USA). The solution was then pH-adjusted to 7.4 by dropwise addition of 1 M HCl while being stirred constantly to avoid precipitation. Milli-Q water was then added to bring the calcein buffer to a final volume of 12.5 mL and the calcein concentration to 50 mM. The resulting MLVs were subjected to eight freeze/thaw cycles using liquid nitrogen and a water bath at 35 °C (five cycles). The MLVs were extruded in a 10 mL stainless steel extruder (Lipex Biomembranes Inc., Vancouver, BC, Canada) at 25 °C to obtain LUVs of 100 nm. For calcein release experiments, the dye-containing vesicles were separated from non-entrapped calcein using a size exclusion chromatographic column (Sephadex G50 Fine, 10 × 150 mm; Sigma-Aldrich, San Louis, MO, USA), eluted with 10 mM Tris-HCl buffer that did not contain calcein. The total lipid concentration of the extruded LUVs was estimated by a colorimetric method as described by Stewart [63].

### 4.9. Isothermal Titration Calorimetry (ITC)

ITC analyses were performed in a Malvern^®^ VP-ITC microcalorimeter (Malvern Panalytical Ltd, Malvern, United Kingdom), at 25 °C. The calibration of the equipment was performed with deionized water Milli-Q^®^ type 1. The solutions were previously degassed in a Microcal Thermovac^TM^ (Malvern Panalytical Ltd, Malvern, UK), accessory from Malvern^®^. Each experiment consisted of 25 successive injections of 5 μL of 20 mM POPG:CL (2:1, mol/mol) LUVs into the calorimetric cell filled with 1.4 mL of peptide solution (25 μM), at 300 s intervals. To eliminate the diffusion effects of material from the syringe to the calorimetric cell, the first injection of 1 μL was discarded. Dilution experiments of LUVs were performed by injections of the samples in 10 mM Tris-HCl, 50 mM NaCl, pH 7.4. The acquisition and isothermal treatments for each analysis were performed using the Microcal Origin 7.0 software for ITC. Non-linear fitting was performed by using the model of one-site binding based on the Wiseman Isotherm [23] (Equation (1)) to obtain the partial molar enthalpy of complexation (= *dQ*/*d*[*X*]) at constant pressure.

(3) $$\bar{{∆}_{comp}H}={\left(\frac{dQ}{d{\left[X\right]}_{tot}}\right)}_{P}={∆}_{int}H{}^{\circ}V_{o}\left[\frac{1}{2}+\frac{1-X_{R}-r}{2\sqrt{(1+X_{R}-r{)}^{2}-4X_{R}}}\right]$$

where *X* is the molar ratio of titrant and M (*X*_R_ = [*X*]*_t_*/[*M*]*_t_*) molar ratio of titrated at any point during the titration. The r parameter is the composition variable (*r* = 1/[*M*]_t_.K_app_) and the *∆_int_H*°, *V_0_* and K_eq_ parameters are the standard complexation enthalpy, effective volume of the solution in the titration cell, and apparent equilibrium constant, respectively.

### 4.10. Dynamic Light Scattering (DLS) 

The changes of hydrodynamic diameter (*D_h_*) and zeta potential (*ζ*P) of POPG:CL (2:1, mol/mol) LUVs and or POPG:CL:LysylPOPG (2:2:1) with addition of the peptide solutions were measured at 35 °C on a Z98 Zeta sizer nano ZS Malvern^®^ model BI-900 particle analyzer (Malvern Panalytical Ltd, Malvern, United Kingdom), The light intensity of the monochromatic laser (4 mW Ne laser, λ of 633 nm) scattered was detected at a 90° angle. The experiments were collected in triplicate on independent experiments using 10 samples of 100 μM LUVs solution containing different peptide concentration with a final volume of 800 μL. For all samples we used 1 mM peptide and 400 μM LUVs stock solution, both suspended in 10 mM Tris-HCl, 50 mM NaCl, pH 7.4. Sample compositions are detailed in Table 5. The samples were incubated at 35 °C for 30 min before reading the hydrodynamic diameter and zeta potential. 

### 4.11. Calcein Release 

Calcein release experiments were carried out in polystyrene microplates (128 × 86 × 14.5 mm) for fluorescence emission measurement in media containing LUVs suspension (100 μM) of POPG:CL (2:1), POPG:CL:LysylPOPG (2:2:1) or POPC:Chol (2:1), different volumes of peptide (250 μM), and buffer (10 mM Tris-HCl, 50 mM NaCl, pH 7.4) solutions to a final volume of 300 μL as detailed in Table 6. 

The increase in CF fluorescence as a function of time at 25 °C was recorded every 1 min in the Spectra Max^®^Paradigm detection platform (Molecular Devices, LLC, Sunnyvale, CA, USA) at excitation and emission wavelengths of 480 and 520 nm, respectively. The stability of the LUVs was monitored for 5 min before the peptide was added. The calcein release after the addition of the peptide was recorded for 15 min. The total calcein released from the LUVs (100%) was determined in a similar experiment using 0.1% Triton-X 100 in the absence of the peptide. Calcein leakage was calculated using Equation (2):

(4) $$Dye\;leakage\left(\%\right)=\frac{\left(F-F_{0}\right)}{\left(F_{T}-F_{0}\right)}\times 100\%$$

where *F*_0_, *F*, and *F_T_* denote the basal fluorescence intensity, fluorescence intensity after addition of peptides, and maximum fluorescence intensity obtained after addition of 0.1% Triton X-100, respectively. Tris-HCl buffer was used as negative control. The experiments were performed in triplicate on independent samples of calcein-loaded LUVs. The results are presented as the average with their standard deviations. The observed rate constant of calcein release, k_obs_, was calculated by using a single-exponential fitting of the recorded data of fluorescence intensity as a function of time [46].

### 4.12. Peptide Formulation 

The formulation used for the experiment was prepared according to the Brazilian Pharmacopoeia 6th edition [64]. It consisted of a gel prepared by dissolving methylparaben (Nipagin^®^; Fragon, Curitiba, SC, Brazil) in water heated to 70 °C. Then, under constant stirring on a hotplate with a magnetic stirrer hydroxyethylcellulose (Natrosol^®^; Fragon, Curitiba, SC, Brazil) was added to form the gel. The process involved alternating stirring with resting periods, and the resulting gel had colorless characteristics and a pH of 6. Additionally, sodium metabisulfite was incorporated as an antioxidant to protect peptides from degradation by oxidation. The components and quantities used are described in Table 7.

### 4.13. In Vivo Assays

#### 4.13.1. Animals

Six-week-old male BALB/c mice (Biotério Central da UFMG, Belo Horizonte, MG, Brazil) were used in this study. The animals were kept in polypropylene boxes measuring 30 × 19 × 13 cm in an environment with a controlled temperature of 25 ± 2 °C, 40% humidity, and a 12/12 h light/dark cycle in Bioterio of *Faculdade de Saúde Santa Casa de Belo Horizonte*. All experimental procedures strictly followed the international protocols for laboratory animal management, and the methods were approved by the Laboratory Animal Research Ethics Committee of the *Faculdade de Saúde Santa Casa de Belo Horizonte* (CEUA-Santa Casa: 001-2023).

#### 4.13.2. Murine Model of Non-Surgical MRSA-Infected Wounds

An in vivo model of MRSA USA300 skin infection was conducted according to Lima et al. [38]. Following the induction of general anesthesia (60 mg/kg ketamine + 8 mg/kg xylazine, i.p.), the dorsal hair was removed, and the skin was cleaned with 70% (*v*/*v*) ethanol. Inside the biological security cabin (Veco^®^ Bioseg 18, Campinas, SP, Brazil), 50 μL of a bacterial suspension at 10^8^ CFU/mL was injected subcutaneously into the dorsal area of the animals. After infection, the animals were placed in previously sterilized polypropylene boxes and kept in this environment with access to autoclaved water and feed ad libitum. After 48 h, an open wound/abscess was observed at the injection site.

#### 4.13.3. Treatment of Animals

After MRSA-induced open wound/abscess, the animals were divided into six experimental groups (*n* = 5). These groups were treated topically with the previously formulated gel containing different concentrations of LyeTx I mnΔK (0.25%, 0.50%, or 1.00%). The positive control received 1.00% vancomycin-containing gel. Groups of animals treated topically with saline (untreated control) and base gel (base control) were also included. All animals were treated for three consecutive days with two daily administrations. Twenty hours after the last treatment, the animals were euthanized, the wounds were aseptically removed, weighted, homogenized in saline, and plated on mannitol agar to determine the bacterial load. 

### 4.14. Statistical Analysis

For the interpretation of the results, statistical analyses were conducted. Initially, the normality of the data was assessed using the Shapiro–Wilk test in SPSS Statistics v. 19 software. If the data did not follow a normal distribution, the histogram, median, standard deviation, and kurtosis were used to check the normality of the data. Unidirectional analysis of (one-way ANOVA) was used. Tukey’s post-test was selected for comparisons between all groups, and Dunnett’s test was used for comparisons with the control group (untreated). Results were expressed as mean ± standard deviation and *p*-value ≤ 0.05 was considered to the statistical significance.

## 5. Conclusions

The synthetic peptide LyeTx I mnDK emerges as a potent antimicrobial agent with notable efficacy against MRSA-induced wounds. Rapid bactericidal action, significant anti-biofilm effects, and a membrane damaging mechanism make it a promising candidate for the urgent challenge of MRSA skin infections, in addition to it showing good selectivity to bacterial cells. The peptide demonstrates superior activity over conventional antibiotics like vancomycin and oxacillin, particularly in eradicating MRSA biofilms and action on bacterial membranes. The LyeTx I mnDK maintains effectiveness even in the presence of mimetic membrane containing lysylphosphatidylglycerol (LysylPOPG) modifications, showing its potential in challenging conditions. Furthermore, its indifference in combination with conventional antibiotics and higher resensibilization suggests a potential role in combination therapy, offering a multifaceted approach to combatting MRSA. The peptide’s application in a formulated gel proves effective in treating MRSA-induced non-surgical mice and is unparalleled by the performance of vancomycin. These findings underscore LyeTx I mnΔK as a promising antimicrobial agent for MRSA-associated skin infections. 

## 6. Patents

LIMA, W.G.; BRITO, J.C.M.; COELHO, A.P.G.; RESENDE, J.M.; VERLY, R.M.; LIMA, M. E. “Formulação para aplicação tópica de peptídeo antimicrobiano e seu uso para produzir medicamentos para tratamento de infecções dermatológicas”. 2023, Brazil. Patent: number: BR13202302645, Institution: INPI—Instituto Nacional da Propriedade Industrial. Deposit: December 15th, 2023.

## Figures and Tables

**Table 1:** Minimum inhibitory concentration (MIC) and minimum bactericidal concentration (MBC) of LyeTx I mnΔK and vancomycin against clinical isolates of methicillin-resistant *Staphylococcus aureus* (MRSA).

Microorganism	Antibacterial Activity (µM)			
	LyeTx I mnΔK		Vancomycin	
	MIC	MBC	MIC	MBC
*S. aureus* 11	8	32	1	8
*S. aureus* 29	8	16	1	2
*S. aureus* 130	8	32	1	2
*S. aureus* 366	16	32	1	8
*S. aureus* 524	16	32	1	2
*S. aureus* 526	16	32	1	4
MRSA USA300	8	16	1	2
MIC_50_	8		1	
MBC_50_	32		2	

MIC_50_: minimum inhibitory concentration required to inhibit 50% of MRSA isolates tested; MBC_50_: minimum bactericidal concentration required to inhibit 50% of MRSA isolates tested.

**Table 2:** Range of fractional inhibitory concentration (FIC) and fractional inhibitory concentration index (FICI) of LyeTx I mn∆K in combination with vancomycin or oxacillin against methicillin-resistant *Staphylococcus aureus* (MRSA).

Class	Antimicrobials	FIC		FICI (ΣFIC)	Effect
		LyeTx I mnΔK	Antimicrobial		
Glycopeptide	Vancomycin	1.00	0.50	1.50	Indifferent
β-lactam	Oxacillin	1.00	0.02	1.02	Indifferent

ΣFICI: fractional inhibitory concentration index. The FICI was interpreted as follows: a FICI ≤ 0.5 is considered evidence of synergy. Additive was defined as 0.5 < FICI < 1. A 1 ≤ FICI ≤ 4 was considered an indifferent effect. Antagonism was defined as a FICI > 4 [22].

**Table 3:** Resensitization of methicillin-resistant *Staphylococcus aureus* (MRSA) to vancomycin and oxacillin using a sub-inhibitory concentration (1/4× MIC) of LyeTx I mnΔK for one hour.

Antibacterial	MICs (µg/mL)		Fold Resensitization
	Not Exposed to LyeTx I mnΔK	Exposed to LyeTx I mnΔK	
Vancomycin	1.0	0.5	2
Oxacillin	128	64	2

Fold of resensitization: it is the ratio of the MIC of antibacterial alone divided by the MIC of antibacterial after resensitization with (1/4× MIC) of LyeTx I mnΔK.

**Table 4:** Effect of LyeTx I mn∆K on *k*_obs_ for the leakage of calcein from POPG:CL and POPG:CL:LysylPOPG LUVs.

LyeTx I mn∆K (mM)	POPG:CL				POPG:CL:Lysyl:POPG			
	%Calcein Released	SD *	*k*_obs_ (s^−1^) × 10^−2^	SD * ×10^−4^	%Calcein Released	SD *	*k*_obs_ (s^−1^) × 10^−2^	SD * ×10^−4^
4	28.51	2.50	0.78	5.01	15.98	1.06	0.629	2.23
8	55.11	1.83	1.01	2.82	33.74	1.01	0.615	1.56
18	72.23	1.49	1.19	3.81	67.73	1.30	0.755	1.13
32	79.45	3.82	1.46	4.36	75.17	2.20	0.853	7.10
64	82.17	2.04	2.16	12.5	77.23	1.99	1.225	4.89

* SD—Standard deviation.

**Table 5:** Sample compositions used for hydrodynamic diameter (*D_h_*) and zeta potential (ζP) measurements.

Sample	*V_pep_* (µL)	*V_LUVs_* (µL)	*V_buffer_* (µL)	[LyeTx I mn∆K] (µM)
1	0	400	400	0
2	4	400	396	5
3	8	400	392	10
4	12	400	388	15
5	16	400	384	20
6	24	400	376	30
7	32	400	368	40
8	40	400	360	50
9	50	400	350	62.5
10	60	400	340	75

*V_pep_* = volume of 1 mM peptide stock solution, *V_LUVs_* = volume of 400 μM POPC:POPG LUVs stock solution, and *V_buffer_* = volume of 10 mM Tri-HCl buffer solution (pH 7.4).

**Table 6:** Sample compositions used for calcein release measurements.

Sample	*V_pep_* (µL)	*V_LUVs_* (µL)	*V_buffer_* (µL)	[LyeTx I mn∆K] (µM)
1	0	150	150	0
2	5	150	145	4,2
3	10	150	140	8,3
4	20	150	130	16,7
5	40	150	110	33,3
6	80	150	70	66,7

*Vpep* = volume of 2 mM peptide stock solution, *V_LUVs_* = volume of 200 μM POPC: POPG LUVs stock solution, and *V_buffer_* = volume of 10 mM Tri-HCl buffer solution (pH 7.4).

**Table 7:** Components used for the formulation of LyeTx I mnΔK.

Component	Concentration
Hydroxyethylcellulose (Natrosol^®^)	2.2%
Sodium metabisulfite	0.6%
Methylparaben (Nipagin^®^)	0.2%
Distilled water	q.s.

q.s.: sufficient quantity to complete the given volume.

## Data Availability

Data are contained within the article and Supplementary Materials.

## Supplementary Materials

The following supporting information can be downloaded at: https://www.mdpi.com/article/10.3390/antibiotics13030248/s1, Figure S1: Isobolograms of combination of peptide LyeTx I mn∆K with vancomycin (A) and oxacillin (B) against methicillin-resistant Staphylococcus aureus (MRSA).; Figure S2: Effect of [LyeTx I mn∆K] on phospholipid membranes as assessed by calcein release from POPC:CL (2:1, mol:mol) at 100 µM of lipid concentration. (a) Time dependence of membrane permeability with LyeTx I mn∆K and (b) percentage of calcein release compared to what was observed after addition of 2% Triton X-100 (positive control, considered as 100%). Data are representative of three independent experiments; Figure S3. Histrogram of size distribution of 200 μM POPG:CL (2:1 mol/mol) in the presence of 30 mM of LyeTx I mn∆K.
