# Supplementary material for: The Synthetic Peptide LyeTx I mn∆K, Derived from Lycosa erythrognatha Spider Toxin, Is Active against Methicillin-Resistant Staphylococcus aureus (MRSA) In Vitro and In Vivo
Source: Antibiotics (Basel). 2024 Mar 8;13(3):248. doi: 10.3390/antibiotics13030248 (PMC10967519; doi:10.3390/antibiotics13030248)
Supplement: Supplementary file 1 [file antibiotics-13-00248-s001.zip › antibiotics-2869536-supplementary.pdf]

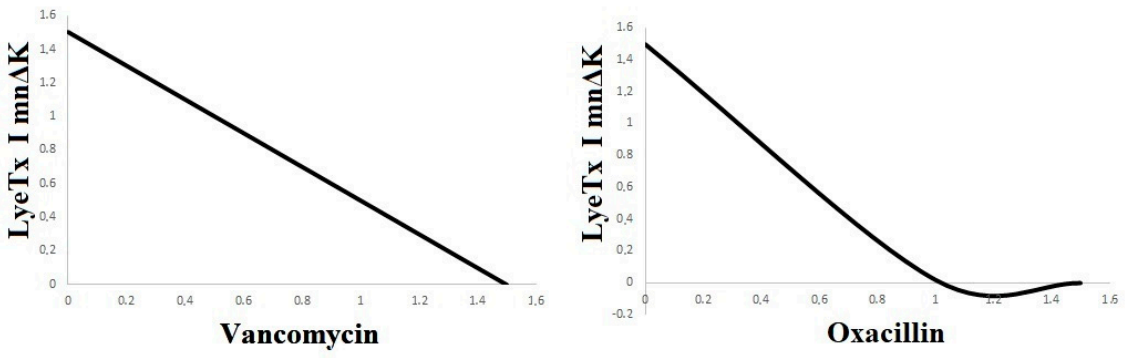

Figure S1. Isobolograms of combination of peptide LyeTx I mnΔK with vancomycin (A) and oxacillin (B) against methicillin-resistant *Staphylococcus aureus* (MRSA).

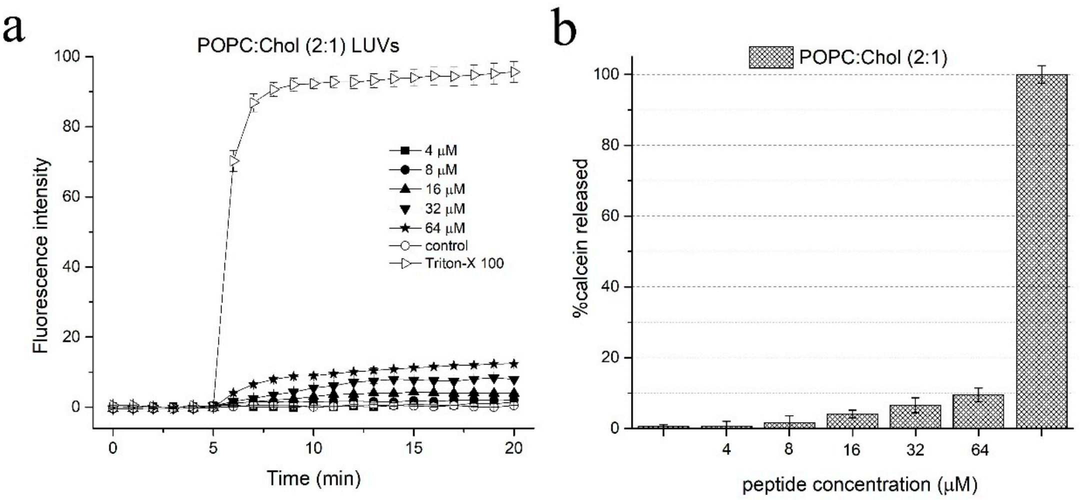

Figure S2. Effect of [LyeTx I mnΔK] on phospholipid membranes as assessed by calcein release from POPC:CL (2:1, mol:mol) at 100 μM of lipid concentration. (**a**) Time dependence of membrane permeability with LyeTx I mnΔK and (**b**) percentage of calcein release compared to what was observed after addition of 2% Triton X-100 (positive control, considered as 100%). Data are representative of three independent experiments.

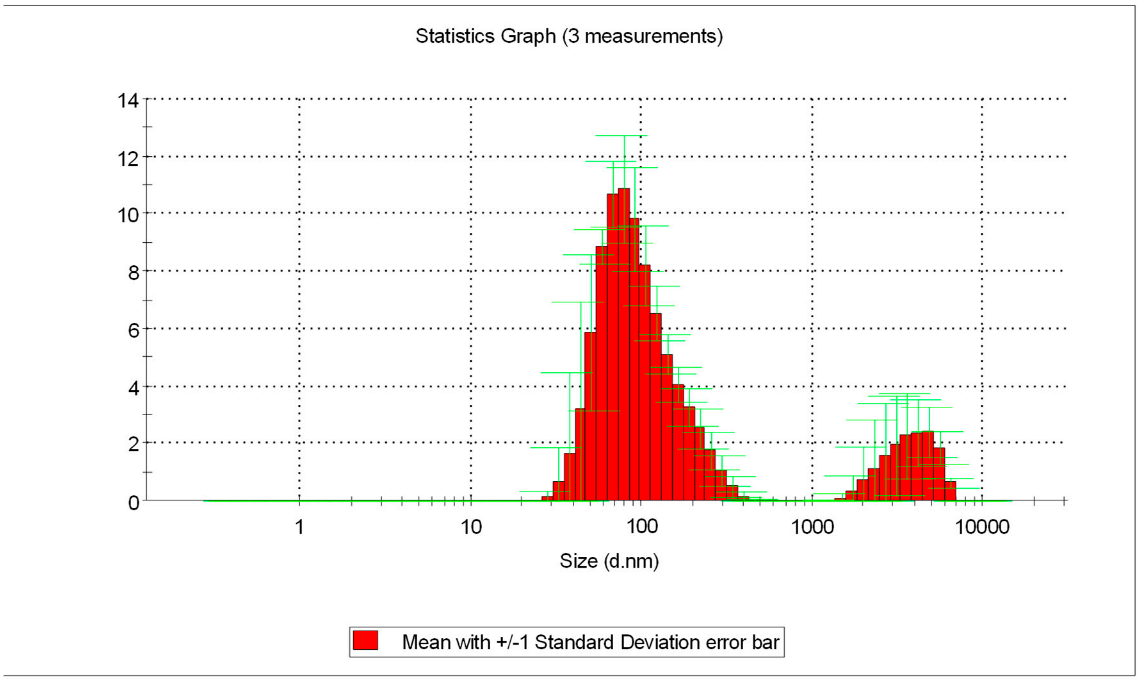

Figure S3. Histrogram of size distribution of 200 μM POPG:CL (2:1 mol/mol) in the presence of 30 mM of LyeTx I mnΔK
